# Chronic Pulmonary Aspergillosis: Disease Severity Using Image Analysis and Correlation with Systemic Proinflammation and Predictors of Clinical Outcome

**DOI:** 10.3390/jof7100842

**Published:** 2021-10-07

**Authors:** Shiang-Fen Huang, Chia-Chang Huang, Kun-Ta Chou, Yu-Jiun Chan, Ying-Ying Yang, Fu-Der Wang

**Affiliations:** 1 Division of Infectious Disease, Department of Medicine, Taipei Veterans General Hospital, Taipei 112201, Taiwan; fdwang@vghtpe.gov.tw; 2 School of Medicine, National Yang-Ming Chiao-Tung University, Taipei 112304, Taiwan; hbjoue@vghtpe.gov.tw; 3 Division of Endocrinology and Metabolism, Department of Medicine, Veterans General Hospital, Taipei 112201, Taiwan; cchuang7@vghtpe.gov.tw; 4 Division of Clinical Skills Training, Department of Medical Education, Taipei Veterans General Hospital, Taipei 112201, Taiwan; yangyy@vghtpe.gov.tw; 5 Department of Chest Medicine, Taipei Veterans General Hospital, Taipei 112201, Taiwan; 6 Division of Microbiology, Department of Pathology and Laboratory Medicine, Taipei Veterans General Hospital, Taipei 112201, Taiwan; yjchan@vghtpe.gov.tw; 7 Department of Medicine, Institute of Clinical Medicine, National Yang-Ming Chiao-Tung University, Taipei 112304, Taiwan

**Keywords:** chronic pulmonary aspergillosis, inflammation, parenchyma analysis

## Abstract

(1) Background: The presentation of chronic pulmonary aspergillosis (CPA) ranges from single granuloma to fibrosis in the affected lung. CPA can be divided into five categories according to European Respirology Society (ERS) guidance but is usually assessed by clinical physicians. Computer-based quantitative lung parenchyma analysis in CPA and its correlation with clinical manifestations, systemic inflammation, and angiogenesis have never been investigated. (2) Method: Forty-nine patients with CPA and 36 controls were prospectively enrolled. Pulmonary function tests (forced vital capacity (FVC), forced expiratory volume in one second (FEV_1_), and FEV_1_/FCV) and biomarkers in the peripheral blood (the chemokines interleukin (IL)-1B, IL-6, IL-10, IL-8, CRP, ESR, MMP1, MMP7, MMP8, TNF-α, calprotectin, SDF-1α, and VEGFA) were measured before antifungal treatment. The disease severity was categorized into mild, moderate, and severe based on chest computed tomography (CT) images. The oxygen demand and overall mortality until the end of the study were recorded. Quantitative parenchyma analysis was performed using the free software 3Dslicer. (3) Results: The results of quantitative parenchyma analysis concorded with the visual severity from the chest CT, oxygen demand, FVC, and FEV_1_ in the study subjects. The decrease in kurtosis and skewness of the lung density histograms on CT, increase in high attenuation area (HAA), and reduced lung volume were significantly correlated with increases in the PMN %, CRP, IL-1B, SDF-1α, MMP1, and Calprotectin in peripheral blood in the multivariable regression analysis. TNF-α and IL-1B at study entry and the CPA severity from either a visual method or computer-based evaluation were predictors of long-term mortality. (4) Conclusion: The computer-based parenchyma analysis in CPA agreed with the categorization on a visual basis and was associated with the clinical outcomes, chemokines, and systemic proinflammation profiles.

## 1. Introduction

*Aspergillus* is a widespread fungus that can lead to detrimental sequelae. The pulmonary manifestation of *Aspergillus* infection ranges from allergic reactions, single *Aspergillus* granuloma, and tracheobronchitis to angio-invasive infection. Such patients may present with chronic infection involving the parenchyma and pleura. Typically, chronic pulmonary aspergillosis (CPA) is defined as fibrobronchiectasis in the lung parenchyma with pleural thickening, with or without fungal balls in a cavity (CCPA) and can be interchanged with subacute invasive aspergillosis (SIA) or necrotizing pneumonia (CNPA) or progress to fibrosis (CFPA) without recovery and with a poor survival probability. Chronic *Aspergillus* infection can progress to systemic invasion in hosts, especially in those that are extremely immunosuppressed [1].

Computer-based lung parenchyma analysis has been widely used to estimate lung volume and disease severity with fibrosis [2] either in chronic obstructive pulmonary disease (COPD) or in interstitial lung disease (ILD) [3,4]. For example, in pulmonary fibrosis (PF), the result of texture analysis of the lung parenchyma is related to disease severity, quality of life, and overall survival [4]. 

The texture of the lung parenchyma is also correlated with systemic inflammatory biomarkers in PF patients [5] and can be evaluated by means of free software, namely, 3Dslicer© (https://www.slicer.org/, accessed on 1 October 2021), which can automatically or interactively perform lung segmentation, followed by lung parenchyma analysis using a chest imaging platform (CIP, https://chestimagingplatform.org/, accessed on 1 October 2021). The skewness and kurtosis of the lung density histogram, lung attenuation (Hounsfield unit, HU), and lung volume can be automatically analyzed by means of CIP and used to evaluate the severity of IPF [2].

In CPA, the residual lung volume depends on the category of CPA, while it may be irreversible once the disease progresses to CFPA. Patients who present with more than two categories of CPA (CCPA and CFPA) are common, and the severity of CPA cannot be easily recorded. Although a previous investigation described the T helper cytokines within different types of CPA [6], and, although image improvement in response to anti-mold therapy indicated a favorable outcome, knowledge of a comprehensive correlation between lung parenchyma and computerized image analysis, the visual characterization of CPA, inflammatory biomarkers, and clinical outcome in CPA patients is limited.

Once CPA is diagnosed, the long-term survival rates for 1 year, 5 years, and 10 years range from 63–86%, 15–62%, and 47–62%, respectively. Mortality in CPA usually depends on the involved lung area of CPA or hypoxia [7] or the presences of vigilance symptoms [8]. Except for treatment failure due to intolerance or resistance to antifungal therapy, biomarkers such as high C-reactive protein (CRP) [9], low serum albumin level [10], and increasing *Aspergillus* IgG after surgical intervention for CPA [8], have been described as predictors of mortality. Comorbidities with poor outcomes, such as immune compromised status, concurrent infection with pneumonia or non-tuberculosis *Mycobacterium*, old age, male sex, low BMI, use of corticosteroids [9], presence of aspergilloma [7,11], chronic lung disease, and the presence of ILD, have also been described [12].

Deficiency in or dysregulation of antigen presentation, intracellular lysosomal reaction, oxidative stress, or regulatory T and T helper cell responses can lead to uncontrolled aspergillosis [13]. For example [14], patients with Jobs syndrome, also known as signal transducer and activator of transcription 3 (STAT3) deficiency, can manifest with high blood IgE levels and are more susceptible to fungal infection. Long-term antifungal treatment and resection for refractory CPA have been suggested, but emergency azole-resistant *Aspergillus* infection has been reported [15]. 

STAT3 deficiency can be found with a lack of IFN-γ and Th17 responses after in vitro stimulation by *Aspergillus* conidia [16], and IFN-γ prophylaxis can be applied to prevent infection. In patients with chronic granulomatous disease (CGD) who are deficient in NADPH oxidase-dependent phagocytosis, IFN-γ prophylaxis provides little protection against invasive filamentous fungal infections [14]. Lack of CARD9 expression was also linked to impaired proinflammatory cytokines Th1-, Th17-, and Th22 responses upon Aspergillus conidia stimulation, which may underlie predisposition to Aspergillus infection [17].

As a result of pro- and anti-inflammatory activity, pathogen recognition, and the proinflammatory cascade, an anti-inflammatory response to avoid uncontrolled destruction via Th1 and Th17 activity was suggested to be involved in the pathogenesis of CPA [13,18].

Inflammation-mediated angiogenesis is thought to be an underlying mechanism, particularly in the invasive form of *Aspergillus* infection, and it is the sum of pro- and anti-inflammation activity in the affected lung. High or low inflammation is usually determined by the presence of neutropenia in the host [19]. In CPA, neutrophil-mediated inflammation is associated with disease activity [20]. Furthermore, lung fibrosis and collagen deposits are usually observed surrounding fungal hyphae [21]. Biomarkers, such as CRP, ESR, fibrinogen [22], TNF-α, IL-6, IL-8 [23], IFN-γ [24], and IL-1B [25], have been correlated with disease activity or treatment response in pulmonary aspergillosis patients.

Although the CFPA has been suggested as an end stage of CPA, international guidance for image-based severity scoring systems in CPA with overall survival correlations has never been described. The aims of this study were to evaluate the effectiveness of determining disease severity via computer-based analysis in CPA and its relationship with systemic inflammation and angiogenic chemokines and to compare patients without CPA as control group. Since a patient might have more than one image characteristic of CPA, we divided CPA into severity scores according to the visual category per the current guideline [1]. Furthermore, we evaluated quantitative parenchyma analysis profiles and their correlations with visual severity on chest CT, oxygen demand, chemokines in peripheral blood, and mortality.

## 2. Materials and Methods

This was a prospective study conducted from May 2018 to May 2021 in a Tertiary Medical Center in Taiwan. Individuals with pulmonary abnormalities who visited a hospital or presented with symptoms and signs of respiratory tract infection and distress or systemic inflammation, such as weight loss or malaise were enrolled. After a complete clinical history, physical evaluation, and at least two weeks of antibacterial treatment if bacterial infection was impressed, peripheral blood was collected, and serum was obtained prior to initiating antifungal treatment. Individuals with newly diagnosed active tuberculosis, uncontrolled malignancy, and invasive pulmonary aspergillosis were excluded.

Patients with CPA were diagnosed and recorded as described in a previous study [26]. The radiological characteristics on chest CT, including fungal balls, cavities, nodules or granuloma with or without halo sign, bronchiectasis, pleural thickening, lung volume reduction, and consolidation, were recorded for all patients. Briefly, one to four types of CPA were recorded on chest CT by the “visual method”, according to European Respiratory Society guidance [1]: (1) *Aspergillus* granuloma or nodules, (2) SIA or chronic necrotic pulmonary aspergillosis (CNPA), (3) chronic cavitary pulmonary aspergillosis (CCPA), or (4) chronic fibrotic pulmonary aspergillosis (CFPA). 

Severe lung fibrosis with traction bronchiectasis was categorized as fibrobronchiectasis, and the number of involved lungs developing CFPA was also recorded. The severity of CPA was defined as “mild” if the patient had solitary *Aspergillus* granuloma or nodules; “moderate” if the patient had CNPA or CCPA without lobar destruction or lung volume reduction; and “severe” if the patient had CFPA, volume reduction on chest imaging, lobe collapse, and CCPA with more than two lobes of the lung.

The controls were individuals who sought medical help for respiratory tract symptoms without the presence of pulmonary infiltration. Patients with allergic bronchopulmonary aspergillosis (ABPA) (with stimulated IgE for *Aspergillus*, central bronchiectasis and reversibility in the bronchodilator test by spirometry, and culture-positive for *Aspergillus* spp.) or allergic asthma were enrolled and characterized within the control group.

Patients who agreed to enter the study were asked to consent to collection of their peripheral blood and serum, which were stored at −70 °C until further analyses. The white blood cell counts, neutrophil percentage (PMN%), CRP, and ESR were recorded at the time of the peripheral blood collection. Serological biomarkers (detection range), including IFN-γ (1221–50,000 pg/mL), IL-1B (2.44–10,000 pg/mL), IL-6 (9.77–40,000 pg/mL), IL-8 (2.44–10,000 pg/mL), IL-10 (2.44–10,000 pg/mL), MMP1 (7.84–32,100 pg/mL), MMP7 (7.7–30,200 pg/mL), TNF-α (8.54–35,000 pg/mL), CXCL12 (SDF-1α) (17.09–70,000 pg/mL), and VEGFA (5.86–24,000 pg/mL), were measured using the Procarta cytokine profiling kit (Human ProcartaPlex, Panomics, San Diego, CA, USA). 

MMP8 (0.16–10 ng/mL) and calprotectin (1.56–100 ng/mL) were measured according to the manufacturer’s guidance (Elabscience^®^ELISA^®^, Houston, TX, USA). *Aspergillus fumigatus*- and *A. terreus*-specific IgG (ImmunoCAP, Thermo Fisher, Waltham, MA, USA) were measured at study entry. If the patient was treated for community-acquired pneumonia prior to anti-mold therapy, peripheral blood was obtained at least 2 weeks after antibacterial antibiotics. The patients were confirmed to have stable vital signs without shock or bacteremia to avoid the influences of acute inflammation resulting from bacterial infection. If the patient had a history of asthma or ABPA, peripheral blood was collected while acute exacerbation was controlled. To avoid the influence of acute deterioration of lung function, a pulmonary function test was performed if the patient was in a steady state within a 1-month interval of study entry and could tolerate spirometry.

Lung parenchyma analysis was performed using 3Dsclicer software and the function of CIP (https://chestimagingplatform.org/, accessed on 1 October 2021). Briefly, after loading the image dicom files in the “Chest window”, the function “interactive segmentation” was applied, and then quantitative analysis was performed using “parenchyma analysis”. The results were recorded for the whole lung area (Supplement Figure S1). The mean and standard deviation (SD) of the high attenuation area (HAA (%), area of attenuation from −600 to −250 HU of whole lung (%)), mean lung attenuation (HU), kurtosis, and skewness of the lung density histogram, and lung volume (L) were recorded for each patient within 1–3 months prior to entering the study.

For the clinical data, the significance values and statistical tests are described in the text, associated figures, and figure legends. Continuous variables were compared using ANOVA or nonparametric tests, and the results are presented as the means ± SD. Categorical variables were compared by means of the chi-squared or Fisher’s exact tests. Univariate regression analysis was conducted to identify the *r*-square (*r*^2^) and *p* values for indicators related to clinical outcomes. Multivariate regression models with backward selection were applied to identify the most relevant cytokines in response to quantitative lung parenchyma analysis. Survival analysis using the Kaplan–Meier (KM) method was conducted to evaluate the CPA severity and inflammatory biomarkers to predict the long-term survival. *p* values were obtained using the Breslow (generalized Wilcoxon) and log-rank tests. The statistics were calculated by *JMP* (16.0, Cary, NC, USA) and SPSS software (28.0, trial version, Chicago, IL, USA).

## 3. Results

### 3.1. Study Subjects

Overall, 49 patients with CPA and 36 patients in the control group were enrolled in the current study. There was no difference in the sex distribution, smoking history, or use of bronchodilators between the CPA and control groups (*p* > 0.05). At study entry, the rates of increased oxygen demand (58.3% vs. 30.5%), reduced lung volume (26.5% vs. 2.8%), comorbidities with COPD (42.9% vs. 16.7%), and malignancy (34.7% vs. 8.3%) were higher in the CPA group than in the control group (*p* < 0.05). Additionally, the rate of patients with a history of asthma or allergies was higher in the control group (36.1% vs. 6.1%) (Table 1).

**Figure 1:**
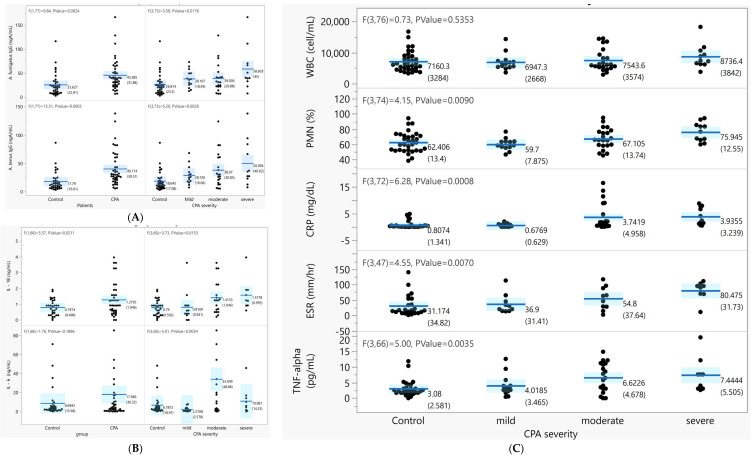
Association between the inflammatory cytokines, *Aspergillus*-specific IgG level, and CPA severity by visual severity. (**A**) The *A. fumigatus* and *A. terreus* IgG levels by disease group and by CPA severity. (**B**) Differences in the peripheral IL-6 and IL-8 levels (pg/mL) between the disease group and CPA severity (*p* < 0.05). (**C**) Clinical laboratory measurement levels by disease group and CPA severity.

We compared the *A. fumigatus* and *A. terreus* IgG levels by visual severity in the CPA and control groups (Figure 1A). The mean levels of both *A. fumigatus* (48.3 ± 34 mgA/mL vs. 27.7 ± 24.6 mgA/mL, *p* < 0.01) and *A. terreus* IgG (44.1 ± 32.5 mgA/mL vs. 21.1 ± 19.8 mgA/mL, *p* < 0.01) were higher in the CPA group than in the control group. Furthermore, the levels of *A. fumigatus* and *A. terreus* IgG were positively correlated with the “visual severity” of CPA (*p* < 0.001), with increasing concentrations in the peripheral blood associated with the increasing severity of CPA.

### 3.2. Quantitative Lung Parenchyma Analysis

#### 3.2.1. Lung Parenchyma Analysis and Pulmonary Function Measurements

In CPA patients, the mean HAA (12.5 ± 7.63%), lung attenuation (−729 ± 69.9 HU), lung volume (3.23 ± 1.09 L), and lung mass (844 ± 188.9 mg) were not different from those in control subjects. The mean kurtosis (5.7 ± 3.6) and skewness (2.19 ± 0.68) were significantly lower than those in the control group (*p* < 0.05). Among CPA patients, the HAA and mean attenuation were significantly increased by visual severity, but the kurtosis, skewness, lung mass, and lung volume were decreased by visual severity (*p* < 0.05, Table 2).

Overall, by univariate regression analysis, the HAA (*r*^2^ = 0.25 and 0.21), kurtosis (*r*^2^ = 0.16 and 0.17), skewness (*r*^2^ = 0.18 and 0.20), and lung volume (*r*^2^ = 0.21 and 0.20) were mildly associated with FVC and FEV1 (*p* < 0.05) but were not relevant to FEV1/FVC (*p* > 0.05). The mean attenuation was borderline correlated with the FVC (*r*^2^ = 0.27, *p* = 0.043) and was not associated with FEV1 (*r*^2^ = 0.21, *p* = 0.008) (Table 3). In CPA patients, the correlation between the pulmonary function test and quantitative parenchyma analysis profiles was more significant, and higher *r*^2^ values (*r*^2^ ranged from 0.40 to 0.52) were observed (Supplement Figure S2).

#### 3.2.2. Lung Parenchyma Analysis and Laboratory Measurements

Overall, in peripheral blood, the cellular distribution, including WBC and platelet counts and the percentage of polymorphic neutrophils (PMN%), were not different between the CPA and control groups. The IL-6 and IL-1B levels in the blood were both significantly higher in the CPA group than in the control group and increased with the severity of CPA (*p* < 0.05, Figure 1B). The PMN%, CRP, and ESR were significantly increased with severity in CPA (*p* < 0.05) (Figure 1C).

To evaluate which cytokines or chemokines were correlated with the texture of the lung parenchyma in CPA patients, univariate regression analysis was performed (Supplement Figure S2B). Overall, the WBC counts, PMN %, CRP, ESR, IL-1B, and IL-8 were generally correlated with the HAA, kurtosis, skewness, and mean lung attenuation in CPA patients (*p* < 0.05).

According to the HAA (Table S1), the kurtosis (Table S2) and skewness (Table S3) were clinically relevant to the severity of CPA, lung function test results, and demand for oxygen. Multivariate regression models with backward selection at the highest *r*^2^ and significant *p* value (*p* < 0.05) were selected. After adjustment for age, the PMN %, TNF-α, IL-1B, SDF-1α, MMP1, and calprotectin were associated with lung fibrosis (kurtosis and skewness) and density (HAA). Chemokines solely related to the PF (kurtosis and skewness) were MMP8 and IL-8 (Table 4).

#### 3.2.3. Lung Parenchyma Analysis and Clinical Outcomes

The correlation between lung parenchyma analysis and oxygen demand is shown in Table 5. In both the control group and CPA patients, the mean HAA was significantly increased with oxygen demand (*p* < 0.05), and the mean attenuation, kurtosis, skewness, and estimated lung volume significantly decreased with high oxygen demand (*p* < 0.05).

**Figure 2:**
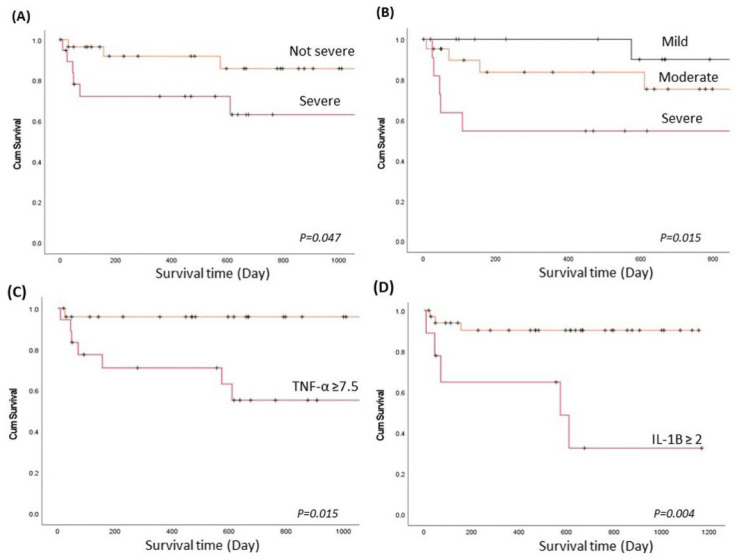
The long-term survival of patients with CPA and prognostic predictors. Survival analysis using the Kaplan–Meier (KM) method to evaluate the CPA severity score and inflammatory biomarkers to predict long-term survival. *p* values were determined using the Breslow (generalized Wilcoxon) test. (**A**). KM plot by severity of CPA using lung parenchyma analysis profiles when the HAA ≥ 13%, skewness < 1.8, or kurtosis < 2.5 (severe), compared to the HAA < 13%, skewness ≥ 1.8, and kurtosis ≥ 2.5 (not severe). (**B**). KM plot by CPA severity using the visual categorization method. (**C**) KM plot by TNF-α level (pg/mL) in peripheral blood at study entry. (**D**) KM plot by IL-1B level (pg/mL) in peripheral blood at study entry.

Long-term follow-up for the 3-year survival was evaluated in the current study. The overall mortality rates were 18.3% (9/49) in the CPA group and 11.1% (4/36) in the control group (*p* > 0.05). In CPA, the IL-1B (2.4 ± 1.03 vs. 1.0 ± 0.8 pg/mL, *p* = 0.001), TNF-α (10.9 ± 4.8 vs. 5.0 ± 3.9 pg/mL, *p* = 0.001), and visual severity (*r*^2^ = 0.19, *p* = 0.026) were higher in patients who died than in surviving patients. Survival analysis using the Kaplan–Meier method showed that patients with earlier mortality presented with higher severity of CPA either by computer-based assessment or by the visual method (*p* < 0.05, Figure 2A,B). Moreover, patients with CPA had a poor prognosis if the blood IL-1B level was higher than 2 pg/mL or if the TNF-α level was higher than 7.5 pg/mL at study entry compared to those with lower IL-1B and TNF-α levels in the blood (*p* < 0.05, Figure 2C,D).

## 4. Discussion

In CPA, the disease severity and imaging characteristics are usually difficult to define. In the current study, we demonstrated that quantitative parenchyma analysis profiles were associated with clinical manifestations and outcomes in CPA patients and had several advantages. 

(1) Conventionally, the severity and characteristics were defined by radiologists or pulmonologist via “visual characterization”, and inter- and within-investigator bias might be present and need to be normalized. The quantitative results were computerized calculations and had diminished human error. 

(2) The categories of CPA can be divided into five types, and one patient could present with more than two types of CPA or could interchange between two types. It is usually difficult to define the “severity” on chest CT if the patient has more than one type of CPA. In the current study, we performed computer-based measurements that calculated universal scores in the whole lung, including the lung density (attenuation), fibrosis (kurtosis and skewness), and lung volume, to evaluate the severity of CPA. The results were consistent with the “visual severity” of CPA. 

(3) The parenchyma analysis profiles were significantly associated with clinical indicators, such as WBC counts, PMN%, and CRP, which means that the parenchyma analysis profiles could be considered an indicator for proinflammation in CPA. 

(4) The parenchyma analysis profiles agreed with pulmonary function tests by spirometry [FEV1, FVC] and oxygen demand, which means that the profiles were relevant to the clinical severity and hypoxia in the study patients. 

(5) We observed that the lung density and fibrosis were related to the overall mortality in CPA and that the higher the HAA and the lower the kurtosis or skewness score was, the higher the mortality would be. Additionally, both increased lung density and fibrosis were relevant to increased oxygen demand in CPA. In summary, the results suggested that computer-based studies could provide an alternative method to help evaluate pulmonary function in patients, especially those who were unable to incorporate spirometry.

In CCPA, the Th2 response is the main immune reaction, and the cytokines IL4, IL5, IL-15, TNF-α, and IL-10 were increased in peripheral blood [27]. One study showed that IL-1B in peripheral blood was associated with disease activity in CPA, and disease activity was evaluated by visual methods and by changes in the CCPA in chest images [25]. One case series showed that the administration of IFN-γ had a protective role in preventing invasive fungal infection; however, its downstream cytokine expression was not comprehensively evaluated [24]. Some research focused on the molecular level, especially in invasive aspergillosis. For example, galactosaminogalactan (GAG) can trigger polymorphologic neutrophil (PMN) apoptosis [28] and chemotaxis [29]. GAG can result in fungal resistance to NADPH-mediated neutrophil killing and against neutrophil-extracellular traps (NETs) [30]. 

GAG suppresses Th1 and Th17 cytokines by inducing the IL-1 receptor antagonist (IL-1Ra) [31] and increasing the Th2 cytokine response [6]. Other molecules, such as β-glucan of *Aspergillus*, can recruit CXCL-1/CXCL-2-dependent neutrophils via the IL-1a/IL1R axis [32]. Th1- and Th17-mediated immune responses and early innate immunity via neutrophil recruitment and killing processes are thought to have protective roles in noninvasive *Aspergillus* infection. 

Our investigation was compatible with previous observations, especially the cytokines IL-6 and IL-1B, which were both significantly higher in the CPA group than in the control group. We also demonstrated that systemic proinflammation was associated with disease severity and that PMN %, ESR, CRP, TNF-α, IL-6, IL-8, MMP1, and MMP8 in peripheral blood increased with the severity of CPA in the univariate or multivariate analysis, consistent with previous investigations [22,23].

Our finding was consistent with the pathogenesis of PF [33] and the notion that SDF-1α is a biomarker for PF that can also be found in CPA. After adjustment for proinflammatory cytokines in the multivariate regression analysis, SDF-1α was consistently correlated with lung fibrosis and density indicators. Aside from angiogenesis directly mediated by *Aspergillus* spp. [19], inflammation and hypoxia can both result in angiogenesis in nonneutropenic patients. In our study, patients with CPA were suggested to have hypoxia-associated angiogenesis, and the chemokine VEGFA was slightly associated with skewness (*r*^2^ = 0.07, *p* = 0.08) and kurtosis (*r*^2^ = 0.08, *p* = 0.07) in the univariate analysis (data not shown). Further investigation regarding hypoxia and angiogenesis in CPA is suggested.

In the era of the COVID-19 pandemic, invasive pulmonary aspergillosis (IPA) has been shown to develop after COVID-19 infection, and the global impact of *Aspergillus* infection indicates that this is an important issue, particularly for patients with underlying pulmonary disease [34]. CPA belongs within a spectrum of diseases that occur after *Aspergillus* infection with immune deficiency. In primary immune deficiency syndromes [13,14,16,17,34], a lack of a proinflammatory reaction and dysfunction of T helper and T regular cells can lead to suboptimal IFN-γ or TNF-α expression. 

As a result of deficient phagocytosis by macrophages and neutrophils, patients may suffer from recurrent bacterial and fungal infection. Long-term antifungal therapy is suggested in this population, but an emergency in azole-resistant *Aspergillus* infections was reported [15]. Immune reconstruction via IFN-γ supplementation [14], stem cell transplantation [35], or GM-CSF therapy [36] may benefit these patients. Therefore, early diagnosis with primary immune deficiency in patients with CPA or with recurrent fungal infection is essential.

However, most CPAs are in patients with acquired immune deficiency. For example, patients with old age [7] or liver cirrhosis [37,38] usually presented with dysregulated in cellular immunity, were with increased morbidities and mortality for *Aspergillus* infection [13]. Among patients with underlying autoimmune disorders, such as SLE [39], with hematological malignancies undergoing intensive chemotherapy, or with HIV infection, profound neutropenia [40] and CD4 T-cell suppression [41] lead to uncontrolled invasive aspergillosis and high mortality, despite appropriate antifungal therapy.

Structural lung diseases, such as COPD, emphysema, and previous *Mycobacterium tuberculosis* (MTB) or NTM infection, were reported to have a high incidence and mortality in CPA [7,42]. In COPD, the dysregulate neutrophil extracellular trap (NET) formation, modulated by M1 or M2 type macrophages [43], and control of *Aspergillus* infection via NETs and macrophages is fundamental [44]. This might explain the higher risk of CPA in COPD and the higher prevalence and acute exacerbation [10]. The use of corticosteroid further compromises the immunity and leads to higher mortality in COPD concurrent with CPA [45]. Another example is MTB and NTM infection, in which PD-1 [46,47] and/or TIM-3 -mediated T-cell exhaustion [48] are considered to be predisposing factors to CPA and poor prognosis [18]. 

Blockage of TIM-3 might provide benefits to adjust cellular immunity against MTB [48]. Another concern in *Mycobacterium* and CPA coinfection is the drug–drug interaction between rifamycin and triazole. Treatment failure in CPA due to discontinuation of rifamycin has been reported [45]. Since most patients with CPA receive long-term antifungal therapy, priority in treating MTB first, followed by CPA or treatment with NTM simultaneously, needs to be individually considered. New-generation antifungal agents without cytochrome P450 metabolism need to be included in future guidelines [49].

In the current study, we first demonstrated that TNF-α and IL-1B were predictors of poor prognosis in CPA. These two cytokines are involved in macrophage activity [50] and the pathogenesis of *Aspergillus* infection [51]. We also provide the first method to evaluate disease severity by computer-based and visual methods, and found that severity correlates with pulmonary function, oxygen demand, systemic proinflammatory biomarkers, and clinical outcomes. Our results are consistent with those of previous investigations, such as the presence of lung fibrosis [12], extent of involved lungs, and hypoxemia status [7], which are related to the clinical outcome. 

The development of a convenient scoring system for disease severity is warranted to define patients with a high risk for poor prognosis and those who would benefit from early initiating multidiscipline antifungal approach. The limitation of this study is its small sample size, single-center design, and lack of a longer follow-up period. Further investigation exploring the impact of different severities and activities of CPA, biomarkers and their changes on imaging and a long-term prognosis are warranted.

## 5. Conclusions

Quantitative computer-based lung parenchyma analysis in CPA was shown to be an effective method for disease severity evaluation and was significantly correlated with the clinical presentations, overall mortality, oxygen demand, and peripheral chemokine levels of systemic proinflammation and PF.

## Figures and Tables

**Table 1:** Clinical characteristics of the study patients.

	Controls		CPA Patients				
	Total (*N* = 36)	*p* *	Total (*N* = 49)	Mild (*n* = 15)	Moderate (*n* = 22)	Severe (*n* = 12)	*p* ^$^
Sex		0.48					0.1
Male	16 (44.4)		33 (68.3)	8 (53.3)	16 (72.7)	9 (75.0)	
Female	20 (55.6)		16 (33.3)	7 (46.7)	6 (27.3)	3 (25.0)	
Age (mean ± SD)	67.0 ± 17.6	0.0002 ^	79.1 ± 12.1	79.7 ± 10.1	77.6 ± 13.6	84.4 ± 12.3	0.69 ^
Oxygen demand		0.001					<0.001
Room air	25 (69.4)		21 (43.8)	13 (86.7)	8 (36.4)	0	
Nasal oxygen ^^^	8 (22.2)		12 (25.0)	2 (13.3)	6 (27.3)	4 (33.3)	
Respiratory failure	3 (8.3)		16 (33.3)	0	8 (36.4)	8 (66.7)	
Smoking		0.2					0.2
Nonsmoker	27 (75.0)		26 (53.1)	10 (66.7)	10 (45.5)	6 (50)	
Ex-smoker	8 (22.2)		22 (44.9)	5 (33.3)	11 (50.0)	6 (50)	
Current smoker	1 (2.8)		1 (2.0)	0	1 (4.5)	0	
Use of Bronchodilator		0.8					0.6
No	20 (55.6)		25 (51.0)	9 (64.3)	13 (59.1)	2 (16.7)	
Yes	16 (44.4)		24 (49.0)	5 (35.7)	9 (40.9)	10 (83.3)	
Presences of CPA							0.09
Aspergilloma	NA		4 (8.2)	2 (13.3)	1 (4.5)	1 (8.3)	0.28
Simple nodules	NA		14 (28.6)	5 (33.3)	8 (36.4)	1 (8.3)	0.04
SIA or CNPA	NA		19 (38.8)	7 (46.7)	8 (36.4)	4 (33.3)	0.15
CCPA	NA		22 (44.9)	3 (20.0)	8 (36.4)	11 (91.7)	0.007
CFPA	NA		14 (28.6)	0	4 (18.2)	10 (83.3)	<0.001
Reduced lung volume							
Yes	1 (2.8)	0.015	13 (26.5)	0	2 (13.6)	10 (83.3)	0.037
No	35 (97.2)		36 (73.5)	15 (100)	19 (86.4)	2 (16.7)	
Comorbidities							
Allergy & asthma	13 (36.1)	0.001	3 (6.1)	1 (6.7)	0	0	0.02
Autoimmune	6 (16.7)	0.75	11 (22.4)	4 (26.7)	6 (27.3)	1 (8.3)	0.54
COPD/emphysema	6 (16.7)	0.002	21 (42.9)	4 (26.7)	11 (50)	6 (50)	0.054
Hemodialysis	2 (5.6)	0.3	2 (4.1)	0	2 (9.1)	0	0.35
Diabetes	11 (30.6)	0.57	9 (18.4)	1 (6.7)	5 (22.7)	3 (25)	0.5
Malignancy	3 (8.3)	0.015	17 (34.7)	3 (20)	9 (40.9)	5 (41.7)	0.23
Previous TB	4 (11.1)	0.8	17 (34.7)	4 (26.7)	4 (18.2)	4 (33.3)	0.5

^^^: Oxygen supplied via a nasal delivery canula. *: *p* value of Pearson *Chi*-square test for comparison between CPA and control groups. ^$^: *p* value for the Pearson *Chi*-squared test in CPA for comparisons among CPA patients with different severities. ^: *p* value by the two-sample *t*-test comparing CPA patients and control group, and by ANOVA in CPA patients. *p* values less than 0.05 are highlighted in red.

**Table 2:** Quantitative lung parenchyma analysis profiles of the study subjects.

	Controls	CPA			
			Visual Severity		
	Total (*N* = 34)	Total (*N* = 48)	Mild (*n* = 15)	Moderate (*n* = 22)	Severe (*n* = 11)
HAA (%)	11.3 ± 8.5	12.05 ± 7.63	7.78 ± 3.18	12.7 ± 6.9	16.5 ± 10.4 ^#^
Mean Attenuation (HU)	−737 ± 76.3	−729 ± 69.9	−768.8 ± 50.0	−722.7 ± 71.1	−688 ± 67.5 ^#^
Kurtosis	7.7 ± 4.1	5.7 ± 3.6 ^$^	8.0 ± 2.3	5.5 ± 3.9	3.18 ± 2.37 ^#^
Skewness	2.5 ± 0.71	2.19 ± 0.68 ^$^	2.678 ± 0.38	2.11 ± 0.72	1.72 ± 0.54 ^@^
Lung Mass (mg)	855 ± 264.5	844 ± 188.9	918.8 ± 159	859.5 ± 203	711 ± 128 ^#^
Lung volume (L)	3.4 ± 1.24	3.23 ± 1.09	4.0 ± 0.95	3.17 ± 0.97	2.32 ± 0.70 ^@^

Numbers are presented as the mean ± standard deviation. HAA: high attenuation area; ^$^: *p* value (<0.05) of *t*-test, compare with control group; ^#^: *p* value < 0.05 by ANOVA, ^@^: *p* < 0.01 by ANOVA.

**Table 3:** Univariate regression analysis of the lung function test in FVC and FEV1 in response to quantitative lung parenchyma analysis profiles.

	FVC % Predicted			FEV1% Predicted			FEV1/FVC % Actual		
	Beta	R^2^	*p*	Beta	R^2^	*p*	Beta	R^2^	*p*
HAA (%)	−3.8	0.25	0.0032	−4.1	0.237	0.0047	0.1	0.022	0.42
Mean of attenuation (HU)	−0.27	0.27	0.0023	−0.25	0.210	0.0083	0	0.006	0.67
Kurtosis	18.4	0.166	0.0206	21.3	0.173	0.0181	2.2	0.023	0.40
Skewness	12.58	0.184	0.0142	13.68	0.200	0.0102	−2	0.033	0.31
Lung volume (L)	0.019	0.217	0.0072	0.032	0.202	0.0099	−0	0.017	0.48

Abbreviations: FVC: forced vital capacity, FEV1: forced expiratory volume in one second, HAA: high attenuation area between −600 and −250 HU, and R^2^: R-square value in regression analysis. *p* values less than 0.05 are highlighted in red.

**Table 4:** Multivariate regression model for quantitative lung parenchyma analysis profiles and chemokines in the peripheral blood of CPA patients.

	Lung Parenchyma Analysis Profiles					
	Skewness (m^3^ *)		Kurtosis (m^3^)		HAA (m^3^)	
	*aR*^2^*=* 0.70, *p* = 0.002		*aR*^2^*=* 0.503, *p* = 0.017		*aR*^2^*=* 0.945, *p* = 0.013	
	Beta	*p*	Beta	*p*	Beta	*p*
Age	−0.307	0.049	−0.357	0.063	0.190	0.216
PMN%	−0.623	0.005	−0.524	0.037	0.584	0.009
TNF-α	0.799	0.005	0.666	0.038	−0.533	0.044
IFN-γ			−0.176	0.355		
IL-1B	−0.738	0.005	−0.650	0.034	0.556	0.027
IL-8			−0.459	0.021		
SDF-1α	−0.377	0.025	−0.475	0.038	0.548	0.002
MMP1	−0.744	0.002	−0.357	0.063	0.532	0.011
MMP8	−0.351	0.057				
Calprotectin	−0.393	0.047			0.383	0.025

Abbreviations: PMN: MMP: Matrix metalloproteinase; SDF-1α: Stromal Cell Derived Factor 1*alpha*; IL: Interleukin; TNF-α: Tumor necrosis factor-alpha; VEGFA: vascular endothelium factor A, CRP: C-reactive protein; FEV1: Forced effort volume; FVC: Forced volume capacity; *aR*^2^: adjusted r-square; WBC: white blood cell; and IFN-γ: Interferon-gamma; * The best fitted models (model number shown in Supplement Table S1) with the lowest *p* value, highest *r*^2^ value and variable numbers are demonstrated. *p* values less than 0.05 are highlighted in red.

**Table 5:** Correlation between for oxygen demand and quantitative parenchyma analysis profiles.

	Control				CPA			
	RA	NO	RF	*p*	RA	NO	RF	*p*
HAA (%)	7.9 ± 5.1	20.0 ± 8.3	18.1 ± 14.3	0.0004	7.1 ± 2.7	11.5 ± 3.5	18.2 ± 10.0	<0.0001
Mean attenuation (HU)	−769 ± 48.9	−661 ± 80.9	−665 ± 94.3	0.0002	−779 ± 41.7	−720 ± 3.1	−676.5 ± 79.1	<0.0001
Kurtosis	9.6 ± 3.1	3.3 ± 2.1	2.9 ± 2.2	<0.0001	8.3 ± 2.4	5.0 ± 3.1	3.2 ± 3.2	<0.0001
Skewness	2.8 ± 0.5	1.7 ± 0.4	1.7 ± 0.5	<0.0001	2.7 ± 0.3	2.1 ± 0.5	1.6 ± 0.5	<0.0001
Lung volume (L)	3.8 ± 0.9	2.2 ± 1.3	2.8 ± 1.3	0.0058	4.1 ± 0.8	3.0 ± 0.7	2.3 ± 0.7	<0.0001

Abbreviations: RA: room air; NO: nasal oxygen; RF: respiratory failure; and HAA: high attenuation area. *p* values less than 0.05 are highlighted in red. Numbers are presented as the mean ± standard deviation. *p* values were obtained using general liner regression model method.

## Supplementary Materials

The following are available online at https://www.mdpi.com/article/10.3390/jof7100842/s1: Figure S1: Computer-based parenchyma analysis by 3Dslicer; Figure S2: Regression analysis and plots with the quantitative parenchyma analysis as independent factors and lung function test as dependent factors. Table S1: Multivariate Regression model (MRM) between laboratory measurement and lung parenchyma analysis profiles (HAA) in CPA patients. Table S2: Multivariate Regression model (MRM) between laboratory measurement and lung parenchymal analysis profiles (Kurtosis) in CPA patients. Table S3: Multivariate Regression model (MRM) between laboratory measurement and lung parenchyma analysis profiles (Skewness) in CPA patients.
