# Supplementary material for: Chronic Pulmonary Aspergillosis: Disease Severity Using Image Analysis and Correlation with Systemic Proinflammation and Predictors of Clinical Outcome
Source: J Fungi (Basel). 2021 Oct 7;7(10):842. doi: 10.3390/jof7100842 (PMC8537715; doi:10.3390/jof7100842)
Supplement: Supplementary file 1 [file jof-07-00842-s001.zip › jof-1389252-supplementary.pdf]

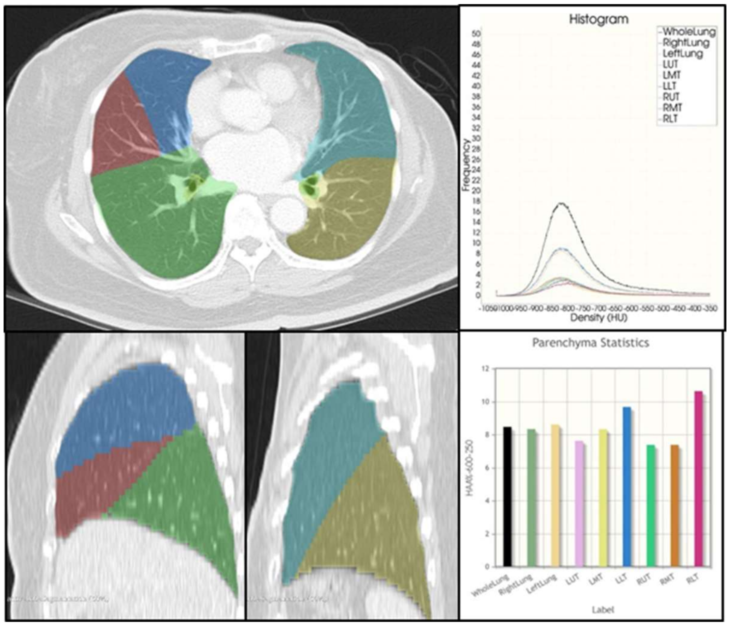

Figure S1. Demographic figure for the result of lung parenchyma analysis by using 3Dslicer. The figure showed interactive lung segmentation for bilateral lung field, and histograms for lung attenuation and HAA (%) for lung attenuation from -600 to -250 HU.

A

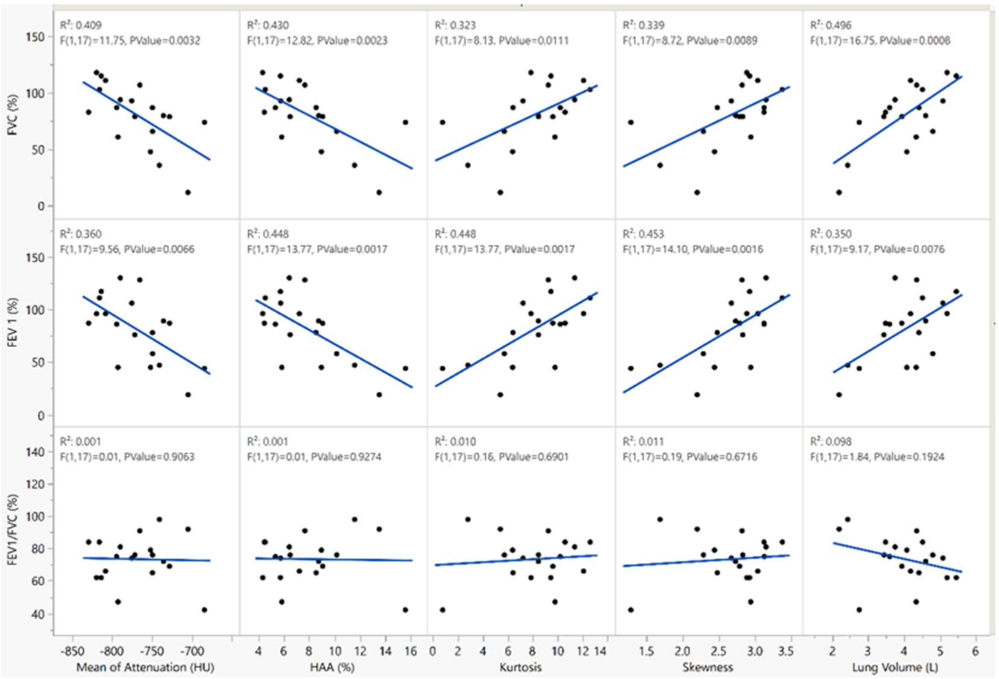

B

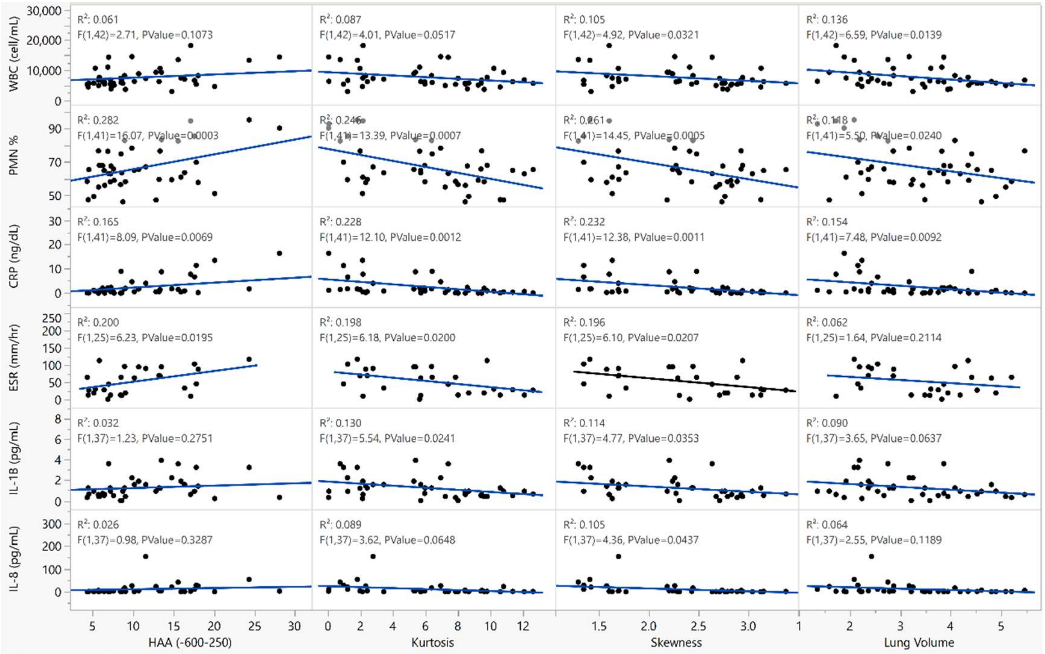

Figure S2. Association between lung volume and quantitative lung parenchyma analysis in CPA patients. **(A)** Univariate regression analysis for the quantitative lung parenchyma analysis as independent variable and lung function test as dependent variable. **(B)** Univariate regression analysis for the quantitative lung parenchyma analysis as independent variables and proinflammation biomarkers [WBC counts, PMN%, CRP, ESR, IL-1B, IL-8] as dependent variable. Only P<0.05 were showed in the figure.

Table S1. Multivariate Regression model (MRM) between laboratory measurement and lung parenchyma analysis profiles (HAA) in CPA patients

Variables	High attenuation area (HAA)							
	model 1 aR^2=0.70, P=0.003		model 2 aR^2=0.68, P =0.002		model 3 aR^2=0.67, P =0.002		model 4 aR^2=0.65, P =0.001	
	Beta	*P*	*Beta*	*P*	*Beta*	*P*	*Beta*	*P*
Age	0.238	0.119	0.202	0.185	0.190	0.216		
PMN%	0.668	0.007	0.694	0.006	0.584	0.009	0.493	0.017
TNF-α	-0.686	0.016	-0.579	0.031	-0.533	0.044	-0.446	0.079
IL-1B	0.532	0.028	0.533	0.032	0.556	0.027	0.529	0.036
IL-6	0.273	0.190						
SDF-1α	0.505	0.013	0.629	0.001	0.548	0.002	0.553	0.002
MMP1	0.675	0.004	0.617	0.007	0.532	0.011	0.491	0.017
MMP7	-0.266	0.160	-0.223	0.240				
Calprotectin	0.495	0.017	0.514	0.015	0.383	0.025	0.338	0.041

The first 4 models were selected using MRM and backward method with highest R^2 and significant P value (<0.05).

Table S2. Multivariate Regression model (MRM) between laboratory measurement and lung parenchymal analysis profiles (Kurtosis) in CPA patients

**Variable**	**Kurtosis**					
	**model 1** aR^2=0.506, *P*=0.034		**model 2** aR^2=0.509 *P* =0.023		**model 3** aR^2=0.503, *P* =0.017	
	Beta	*P*	*Beta*	*P*	*Beta*	*P*
Age	-0.412	0.043	-0.401	0.045	-0.357	0.063
PMN%	-0.706	0.020	-0.599	0.025	-0.524	0.037
TNF-α	0.860	0.022	0.821	0.025	0.666	0.038
IFN-γ	-0.235	0.249	-0.244	0.229	-0.176	0.355
IL-1B	-0.649	0.038	-0.673	0.030	-0.650	0.034
SDF-1α	-0.505	0.026	-0.424	0.034	-0.459	0.021
MMP1	-0.657	0.020	-0.577	0.025	-0.475	0.038
MMP7	0.216	0.359				
Calprotectin	-0.335	0.181	-0.211	0.299		

The first 3 models were selected using MRM and backward method with highest R^2 and significant *P* value (<0.05).

Table S3. Multivariate Regression model (MRM) between laboratory measurement and lung parenchyma analysis profiles (Skewness) in CPA patients

Variable	Skewness					
	**model 1** aR^2=0.70, *P*=0.006		**model 2** aR^2=0.69, *P* =0.003		**model 3** aR^2=0.70, *P* =0.002	
	Beta	*P*	*Beta*	*P*	*Beta*	*P*
Age	-0.365	0.034	-2.243	0.046	-0.307	0.049
PMN%	-0.642	0.012	-3.423	0.006	-0.623	0.005
TNF-α	0.855	0.005	3.412	0.006	0.799	0.005
IL-1B	-0.759	0.006	-3.353	0.006	-0.738	0.005
IL-8	-0.230	0.322				
SDF-1α	-0.443	0.026	-2.636	0.023	-0.377	0.025
MMP1	-0.735	0.005	-3.977	0.002	-0.744	0.002
MMP7	0.213	0.285	0.871	0.402		
MMP8	-0.218	0.294	-1.813	0.097	-0.351	0.057
Calprotectin	-0.312	0.231	-2.353	0.038	-0.393	0.047

The first 3 models were selected using MRM and backward method with highest R^2 and significant *P* value (<0.05).
